# Global Assessment of Retinal Arteriolar, Venular and Capillary Microcirculations Using Fundus Photographs and Optical Coherence Tomography Angiography in Diabetic Retinopathy

**DOI:** 10.1038/s41598-019-47770-9

**Published:** 2019-08-13

**Authors:** Tien-En Tan, Quang Nguyen, Jacqueline Chua, Leopold Schmetterer, Gavin Siew Wei Tan, Chee Wai Wong, Andrew Tsai, Gemmy Chui Ming Cheung, Tien Yin Wong, Daniel Shu Wei Ting

**Affiliations:** 1 Singapore Eye Research Institute, Singapore, Singapore National Eye Centre, Singapore, Singapore; 2 Duke-National University of Singapore Medical School, Singapore, Singapore; 3 Department of Ophthalmology, Lee Kong Chian School of Medicine, Nanyang Technological University, Singapore, Singapore; 4 Department of Clinical Pharmacology, Medical University of Vienna, Vienna, Austria; 5 Center for Medical Physics and Biomedical Engineering, Medical University of Vienna, Vienna, Austria

**Keywords:** Retinal diseases, Diabetes complications, Circulation

## Abstract

Retinal arterioles, venules and capillaries are differentially affected in diabetes, and studying vascular alterations may provide information on pathogenesis of diabetic retinopathy (DR). We conducted a cross-sectional study on 49 diabetic patients, who underwent fundus photography and optical coherence tomographic angiography (OCT-A). Fundus photographs were analysed using semi-automated software for arteriolar and venular parameters, including central retinal arteriolar equivalent (CRAE), central retinal venular equivalent (CRVE) and fractal dimension (FD). Capillary parameters were measured using OCT-A, including capillary density index (CDI) and capillary FD of superficial (SVP) and deep (DVP) vascular plexuses. Severe DR was defined as severe non-proliferative DR and proliferative DR. We found that eyes with severe DR had narrower CRAE and sparser SVP CDI than eyes without. In logistic regression analysis, capillary parameters were more associated with severe DR than arteriolar or venular parameters. However, combining arteriolar, venular and capillary parameters provided the strongest association with severe DR. In linear regression analysis, eyes with poorer visual acuity had lower CRAE and FD of arterioles, venules, and DVP capillaries. We concluded that the retinal microvasculature is globally affected in severe DR, reflecting widespread microvascular impairment in perfusion. Arteriolar, venular and capillary parameters provide complementary information in assessment of DR.

## Introduction

Diabetic retinopathy (DR) ranks among the leading causes of visual impairment and blindness worldwide, especially in the working-age population^1–5^. The risk of developing visual loss from DR has been traditionally determined based on classification of DR severity^6^. On this scale, patients with more severe DR, such as severe non-proliferative DR (NPDR) or proliferative DR, are at higher risk for developing visual loss^6^. If detected in a timely manner, there are effective therapies to halt progression and prevent visual loss in these patients^7,8^.

It is well known that DR is a microvascular complication of diabetes. However, the extent of microvascular involvement in DR is unknown. Retinal arterioles and venules typically measure 15–150 µm in size, while the capillaries measure 5–15 µm^9^. There has been substantial research demonstrating that quantitative retinal arteriolar and venular parameters can be measured from fundus photographs using computer software (such as the Singapore I Vessel Assessment [SIVA]), and that such changes (e.g. narrower arteriolar and venular caliber, and reduced arteriolar fractal dimensions [FD]) are associated with increasing severity and progression of DR^10–26^. Such techniques, however, cannot be applied to retinal capillaries. Until recently, assessment of retinal capillaries has relied primarily on fundal fluorescein angiography (FFA)^27,28^. FFA provides detailed information on the retinal capillary network, but it is time-consuming, invasive, and requires intravenous contrast, which limits its use. The advent of optical coherence tomography angiography (OCT-A) has made rapid, non-invasive assessment of the retinal capillaries possible^29–31^. With OCT-A, detailed images of the retinal capillary network can be acquired and segmented into distinct anatomical layers and plexuses^30,32,33^. To date, numerous studies have investigated the capillary changes (e.g. reduced capillary density and FD measurements) observed with OCT-A in eyes with DR^27,32,34–40^. With further study, such quantitative retinal arteriolar, venular and capillary parameters may have clinical relevance, as potential biomarkers for DR severity, that could assist in monitoring of disease progression or treatment^27^.

It may be that fundus photographs and OCT-A can both provide useful quantitative information in DR, but no study has yet utilized these techniques simultaneously, and in the same eyes. The primary aim of this study was therefore to quantify retinal arteriolar and venular parameters with fundus photograph analysis using SIVA software, and capillary parameters with OCT-A in eyes of diabetic patients, and to investigate the relationship between these parameters and the presence of severe DR. The secondary aims of this study included exploring the relationship between: (1) retinal vascular parameters and visual acuity (VA), and (2) OCT-A capillary parameters and fundus photograph arteriolar/venular parameters.

## Results

Data from 1 patient was excluded because of poor OCT-A scan quality. Therefore, 49 right eyes of 49 patients were used in eventual analyses. Mean (SD) age was 59.2 (8.8) years, and 27 (55%) patients were male. The majority of patients were Chinese (n = 34, 69%), followed by Indian (n = 8, 16%) and Malay (n = 7, 14%), in keeping with local population demographics. In terms of DR severity, there were 9 (18%), 10 (20%), 10 (20%), 8 (16%) and 12 (25%) eyes with no DR, mild NPDR, moderate NPDR, severe NPDR and proliferative DR respectively. Mean (SD) logarithm of the minimum angle of resolution (logMAR) VA in each group was 0.211 (0.127), 0.180 (0.063), 0.200 (0.229), 0.314 (0.234) and 0.327 (0.261) respectively (p-trend = 0.087). There were 29 (59%) eyes with non-severe DR, and 20 (41%) eyes with severe DR. In total, 16 (33%) eyes had diabetic macular edema (DME) within the central 3 × 3 mm zone. Of these, 7 (14%) were in the non-severe DR category, and 9 (18%) were in the severe DR category.

Key retinal vascular parameters at each DR severity level are summarized in Table 1. No significant differences were found in arteriolar and venular tortuosity or branching angles and coefficients between eyes with and without severe DR. Mean (SD) central retinal arteriolar equivalent (CRAE) was higher in eyes with mild NPDR than no DR (157.3 [15.3] vs 143.3 [23.8]), but then was progressively lower from 157.3 (15.3) in mild NPDR, to 134.6 (17.8) in proliferative DR (p-trend = 0.046). Mean (SD) superficial venous plexus (SVP) global capillary density index (CDI) showed progressive reduction from 0.360 (0.021) in no DR to 0.333 (0.010) in proliferative DR (p-trend <0.001). When comparing SIVA parameters between groups, severe DR was associated with a lower mean (SD) CRAE of 135.7 (16.4), compared to 151.3 (19.7) in non-severe DR (p = 0.009). Other SIVA parameters were also reduced in severe DR, but these differences were not statistically significant. For OCT-A capillary parameters, severe DR was associated with lower mean (SD) SVP global CDI of 0.336 (0.016), compared to 0.354 (0.018) in non-severe DR (p = 0.001). Other OCT-A parameters did not show statistically significant differences.

**Table 1:** Retinal arteriolar, venular and capillary parameters at different levels of diabetic retinopathy severity.

DR severity level	Non-severe DR (n = 29)			Severe DR (n = 20)		p-trend (No DR to Proliferative DR)^a^	p-value (non-severe DR vs severe DR)^b^
	No DR (n = 9)	Mild NPDR (n = 10)	Moderate NPDR (n = 10)	Severe NPDR (n = 8)	Proliferative DR (n = 12)		
**Arteriolar and venular (SIVA) parameters**, **mean (SD)**							
CRAE	143.3 (23.8)	157.3 (15.3)	151.4 (20.3)	137.5 (15.1)	134.6 (17.8)	0.046	
	151.3 (19.7)			135.7 (16.4)			0.009
CRVE	219.9 (32.1)	220.2 (18.9)	211.9 (36.6)	213.2 (19.1)	198.1 (30.6)	0.093	
	216.9 (29.3)			204.1 (27.1)			0.163
AVR	0.657 (0.098)	0.715 (0.056)	0.727 (0.128)	0.645 (0.040)	0.686 (0.075)	0.454	
	0.704 (0.100)			0.669 (0.065)			0.123
FDt	1.368 (0.114)	1.349 (0.091)	1.398 (0.086)	1.364 (0.089)	1.300 (0.148)	0.318	
	1.373 (0.094)			1.325 (0.129)			0.219
FDa	1.131 (0.104)	1.151 (0.085)	1.175 (0.072)	1.126 (0.107)	1.093 (0.106)	0.324	
	1.155 (0.084)			1.106 (0.105)			0.150
FDv	1.192 (0.089)	1.138 (0.075)	1.206 (0.077)	1.172 (0.090)	1.134 (0.113)	0.384	
	1.179 (0.082)			1.149 (0.104)			0.282
**Capillary (OCT-A) parameters**, **mean (SD)**							
SVP CDI	0.360 (0.021)	0.356 (0.022)	0.347 (0.009)	0.340 (0.023)	0.333 (0.010)	<0.001	
	0.354 (0.018)			0.336 (0.016)			0.001
DVP CDI	0.360 (0.019)	0.356 (0.025)	0.352 (0.019)	0.346 (0.021)	0.345 (0.021)	0.048	
	0.356 (0.021)			0.345 (0.020)			0.093
SVP FDc	1.515 (0.072)	1.581 (0.049)	1.564 (0.076)	1.546 (0.040)	1.601 (0.050)	0.059	
	1.555 (0.070)			1.579 (0.053)			0.290
DVP FDc	1.537 (0.090)	1.612 (0.060)	1.599 (0.070)	1.556 (0.074)	1.533 (0.125)	0.350	
	1.584 (0.078)			1.542 (0.106)			0.089

Abbreviations: DR = diabetic retinopathy; NPDR = non-proliferative diabetic retinopathy; SIVA = Singapore I Vessel Assessment; CRAE = central retinal arteriolar equivalent; CRVE = central retinal venular equivalent; AVR = arteriole-venule ratio; FDt = total fractal dimension; FDa = arteriole fractal dimension; FDv = venule fractal dimension; OCT-A = optical coherence tomography angiography; SVP = superficial vascular plexus; CDI = capillary density index; DVP = deep vascular plexus; FDc = capillary fractal dimension.

^a^p-trends calculated using the Wilcoxon-type test for trend.

^b^p-values calculated using the Mann-Whitney U test.

In linear regression analysis (Table 2), persons with severe DR tended to have significantly narrower CRAE (β = −16.00, 95% CI: −27.01 to −4.99, p = 0.007) and sparser SVP CDI (β = −0.02, 95% CI: −0.03 to −0.01, p = 0.002). These associations were present even after accounting for the presence of DME, which was included as an additional independent variable in the regression analysis. Logistic regression analysis was also performed to associate vascular parameters with severe DR status (Table 3). Among SIVA arteriolar and venular parameters, CRAE had the highest pseudo-R^2^ value (0.215, p = 0.005). Pseudo-R^2^ value improved to 0.367 when all arteriolar and venular parameters were combined (p = 0.080). Among OCT-A capillary parameters, SVP CDI had the highest pseudo-R^2^ value of 0.377 (p < 0.001). Similarly, combining all capillary parameters produced much better results (pseudo-R^2^ 0.651, p < 0.001). Overall, OCT-A capillary parameters (pseudo-R^2^ 0.651, p < 0.001) were more closely associated with severe DR than SIVA parameters (pseudo-R^2^ 0.367, p = 0.080). The best results, however, were obtained by combining all arteriolar, venular and capillary parameters (pseudo-R^2^ 0.725, p = 0.002).

**Table 2:** Associations of severe diabetic retinopathy status and visual acuity (independent variables) with retinal arteriolar, venular and capillary parameters (dependent variables), using linear regression.

Retinal vascular parameters	Severe DR^a^			LogMAR VA^a^		
	β	95% CI	p-value	β	95% CI	p-value
**Arteriolar and venular (SIVA) parameters**						
CRAE	−16.00	−27.01 to −4.99	0.007	−34.81	−64.15 to −5.46	0.025
CRVE	−14.22	−31.12 to 2.68	0.106	−42.32	−85.56 to 0.92	0.062
AVR	−0.03	−0.08 to 0.02	0.244	−0.02	−0.15 to 0.12	0.798
FDt	−0.05	−0.12 to 0.01	0.125	−0.26	−0.41 to −0.11	0.001
FDa	−0.05	−0.11 to 0.01	0.093	−0.17	−0.30 to −0.05	0.011
FDv	−0.03	−0.09 to 0.02	0.220	−0.22	−0.35 to −0.10	0.001
**Capillary (OCT-A) parameters**						
SVP CDI	−0.02	−0.03 to −0.01	0.002	−0.02	−0.05 to 0.01	0.182
DVP CDI	−0.01	−0.02 to 0.00	0.152	−0.01	−0.04 to 0.02	0.377
SVP FDc	0.02	−0.02 to 0.06	0.304	−0.04	−0.13 to 0.06	0.439
DVP FDc	−0.04	−0.10 to 0.01	0.118	−0.12	−0.23 to −0.02	0.027

Abbreviations: DR = diabetic retinopathy; logMAR = logarithm of the minimum angle of resolution; VA = visual acuity; CI = confidence interval; SIVA = Singapore I Vessel Assessment; CRAE = central retinal arteriolar equivalent; CRVE = central retinal venular equivalent; AVR = arteriole-venule ratio; FDt = total fractal dimension; FDa = arteriole fractal dimension; FDv = venule fractal dimension; OCT-A = optical coherence tomography angiography; SVP = superficial vascular plexus; CDI = capillary density index; DVP = deep vascular plexus; FDc = capillary fractal dimension; DME = diabetic macular edema.

^a^Bivariate linear regression analysis with DME included as an independent variable.

**Table 3:** Pseudo-R^2^ values of logistic regression models for severe diabetic retinopathy based on retinal arteriolar, venular and capillary parameters.

Arteriolar and venular (SIVA) parameters	Pseudo-R^2^ value^a^ for severe DR	p-value^b^	Capillary (OCT-A) parameters	Pseudo-R^2^ value^a^ for severe DR	p-value^b^
CRAE	0.215	0.005	SVP CDI	0.377	<0.001
CRVE	0.065	0.126	DVP CDI	0.084	0.085
AVR	0.063	0.161	SVP FDc	0.042	0.214
FDt	0.060	0.145	DVP FDc	0.069	0.110
FDa	0.083	0.081			
FDv	0.033	0.273			
All arteriolar and venular (SIVA) parameters combined	0.367	0.080	All capillary (OCT-A) parameters combined	0.651	<0.001
All arteriolar, venular and capillary parameters combined				0.725	0.002

Abbreviations: SIVA = Singapore I Vessel Assessment; DR = diabetic retinopathy; OCT-A = optical coherence tomography angiography; CRAE = central retinal arteriolar equivalent; CRVE = central retinal venular equivalent; AVR = arteriole-venule ratio; FDt = total fractal dimension; FDa = arteriole fractal dimension; FDv = venule fractal dimension; SVP = superficial vascular plexus; CDI = capillary density index; DVP = deep vascular plexus; FDc = capillary fractal dimension.

^a^McKelvey and Zavoina’s pseudo-R^2^ value.

^b^p-values from significance of model test.

As a secondary aim of the study, the relationship between retinal vascular parameters and VA was explored with linear regression (Table 2). Persons with higher (worse) logMAR VA tended to have significantly narrower CRAE (β = −34.81, 95% CI: −64.15 to −5.46, p = 0.025) and lower FD of arterioles (β = −0.17, 95% CI: −0.30 to −0.05, p = 0.011), venules (β = −0.22, 95% CI: −0.35 to −0.10, p = 0.001), and capillaries in the deep vascular plexus (DVP) (β = −0.12, 95% CI: −0.23 to −0.02, p = 0.027). Similarly, these associations were present after adjusting for DME as an additional independent variable in the regression analysis.

As another secondary aim, correlations between SIVA and OCT-A parameters were also explored (Table 4). OCT-A DVP capillary FD (FDc) measurements showed fair positive correlation with SIVA combined FD of arterioles and venules (FDt) (r = 0.366, p = 0.012) and arteriolar FD (FDa) (r = 0.411, p = 0.005) measurements. SVP CDI and CRAE were poorly correlated (r = 0.150, p = 0.332).

**Table 4:** Correlation of retinal arteriolar and venular parameters with capillary parameters, across all diabetic retinopathy severity levels.

Capillary (OCT-A) parameters	Arteriolar and venular (SIVA) parameters	Spearman’s correlation coefficient r	p-value
SVP CDI	CRAE	0.150	0.332
	CRVE	0.037	0.813
	AVR	0.055	0.724
DVP CDI	CRAE	0.064	0.678
	CRVE	0.158	0.305
	AVR	−0.167	0.279
SVP FDc	FDt	0.033	0.826
	FDa	0.082	0.590
	FDv	0.008	0.958
DVP FDc	FDt	0.366	0.012
	FDa	0.411	0.005
	FDv	0.267	0.073

Abbreviations: OCT-A = optical coherence tomography angiography; SIVA = Singapore I Vessel Assessment; SVP = superficial vascular plexus; CDI = capillary density index; DVP = deep vascular plexus; FDc = capillary fractal dimension; CRAE = central retinal arteriolar equivalent; CRVE = central retinal venular equivalent; AVR = arteriole-venule ratio; FDt = total fractal dimension; FDa = arteriole fractal dimension; FDv = venule fractal dimension.

## Discussion

This is a novel study that quantified retinal arteriolar, venular and capillary parameters with fundus photographs and OCT-A in the same eyes. Our study adds to the growing body of evidence showing that there are widespread microcirculation changes in eyes with DR. We demonstrate quantitative alterations in retinal arteriolar parameters, principally CRAE, and retinal capillary parameters, which may be useful in global assessment of DR severity. Eyes with more severe DR show diffuse changes in both the larger arteriolar and smaller capillary microvasculature.

In terms of larger arteriolar parameters quantified by SIVA, we found that narrower CRAE was associated with severe DR. This is consistent with other studies using the same semi-automated method of quantification^10,11,15^. In the smaller capillary network assessed by OCT-A, our results demonstrated that lower SVP CDI was associated with severe DR. Our findings are consistent with other OCT-A studies showing reduction in perifoveal vessel density parameters with increasing DR severity^35,38,41,42^. On the other hand, results for capillary FD analysis in the literature have been conflicting, with some studies reporting a decrease in capillary FD with increasing severity of DR, while others report an increase^27,42^. Our study failed to show a statistically significant change in FDc with DR severity.

From a pathophysiologic point of view, both these arteriolar and capillary findings are consistent with our understanding of DR as an ischemic process. It also follows that reduction in retinal arteriolar caliber with increasing severity of disease, would result in reduction in capillary density. However, in our correlational analysis between arteriolar and capillary parameters, it was interesting that CRAE and SVP CDI correlated poorly (r = 0.150, p = 0.332). Examination of the values at each level of DR severity revealed that SVP CDI showed a progressive linear reduction from no DR to proliferative DR, while CRAE showed an initial increase from no DR to mild NPDR, followed by a linear reduction from mild NPDR to proliferative DR (Table 1). This discordance was likely to be the reason for the poor correlation between the two parameters. In fact, this pattern observed with CRAE is interesting because it is consistent with some other reports in the literature, and may provide insight into the pathophysiology of DR in its early stages. Some prospective studies have shown that an increase in CRAE is associated with incident DR, and postulate that this may be an early sign reflecting problems with arteriolar autoregulation dystfunction^26,43^. Others studies have shown that in the early stages of DR, hyperglycemia and hypoxia induce retinal vasodilation, leading to initial hyperperfusion^44–46^.

Our logistic regression models showed that OCT-A capillary parameters were more closely associated with severe DR than arteriolar and venular parameters from SIVA analysis (Table 3). Clearly, eyes with more severe DR have more extensive capillary dropout, which is better appreciated on OCT-A. Our clinical classification of DR severity is based on the risk of progression to neovascular complications. It would follow that the degree of capillary ischemia is closely related to neovascularization. Nevertheless, the strongest association with severe DR in logistic regression analysis was obtained when combining all retinal arteriolar, venular and capillary parameters. This reinforces the point that DR affects the retinal vasculature globally, from the larger caliber arterioles and venules, down to the smaller retinal capillaries. Quantitative assessment of the retinal microvasculature with fundus photograph analysis and OCT-A seem to provide different, but complementary information, and both are important in global assessment of eyes with DR.

As a secondary aim of the study, we also explored the relationship between SIVA arteriolar/venular parameters, and OCT-A capillary parameters in the same eyes. In correlation analysis, we demonstrated that arteriolar FD measured by SIVA analysis correlated with capillary FD of the DVP on OCT-A. To our knowledge, this is a novel finding showing correlation between retinal arterioles and capillaries in the same eyes.

We also explored the relationship between retinal vascular parameters and VA, and demonstrated that lower arteriolar, venular and capillary FD were associated with poorer VA. Fractal dimension is a quantitative parameter that represents the degree of complexity of the retinal vascular tree^47^. The mechanism by which fractal dimensions are affected in DR is still unclear. It is postulated that impaired retinal vascular autoregulation and collateral formation in response to hypoxia result in decreased complexity of the retinal vascular tree^17^. Why lower FD was correlated with poorer VA in our study is unclear. Perhaps reduced complexity of the retinal vasculature is a manifestation of macular ischemia. Regardless of mechanism, it seems that eyes with DR show global changes in fractal dimensions from the larger retinal arterioles and venules, down to the smaller capillary level, and future work in this area may expand our understanding of the pathophysiology of the disease. We would also like to acknowledge that VA can be affected by numerous other factors, such as cataracts, media opacity, uncorrected refractive error and age. In fact, even short-term fluctuation in refractive error due to osmotic swelling of the crystalline lens in diabetics with transient elevation of blood glucose levels can be a significant cause of poor VA. Therefore, our findings in relation to VA in this study are interesting, but need to be interpreted with a measure of caution.

The other limitations of this study must also be clearly acknowledged. First, the cross-sectional nature of this study prevents us from drawing conclusions about causation, and we are unable to investigate how these retinal vascular parameters affect DR progression. Also, our modest sample size meant that some of our comparisons failed to achieve statistical significance, which limits our ability to draw conclusions in these areas. Hypertension, hyperlipidemia, and other systemic diseases can also affect arteriolar and venular parameters, such as CRAE or central retinal venular equivalents (CRVE), and capillary parameters, such as CDI and FD^27,48^. Therefore, our results could have been confounded by co-morbid disease in our patients, and the magnitude of this effect is uncertain.

Finally, while OCT-A has come a long way, the technology is still far from mature. OCT-A measurements in this study were performed with swept source OCT-A (DRI OCT Triton; Topcon Corporation, Tokyo, Japan), using the angiography ratio analysis method. Numerous other OCT-A devices are commercially available, each with their own proprietary algorithms for vessel quantification and analysis. Studies have shown that OCT-A measurements are not comparable between different devices^49^. Interpretation of OCT-A images can also be confounded by image artifacts, particularly in deeper retinal layers^27,50^. Also, OCT-A parameters in this study were derived by segmentation into SVP and DVP. However, a middle capillary plexus has also been demonstrated on histological studies, and some groups advocate further segmentation of OCT-A images into superficial, middle and deep layers^32,33,41^. This middle plexus resides in the inner plexiform layer (IPL), and in our study, would have been included within the SVP. This may have confounded our results, particularly for SVP parameters.

Further studies need to be performed to see if the retinal vascular changes we demonstrate in severe DR are reversible. If so, these parameters may be useful as biomarkers for DR severity, and may have a role in clinical trials assessing therapies for DR, to demonstrate changes in retinal perfusion. Longitudinal studies could also explore the relationship between retinal vascular parameters and risk of progression to proliferative complications or visual loss. Finally, the methods of image analysis used in this study were all semi-automated. Artificial intelligence and deep learning systems have demonstrated great potential for fully-automated analysis of fundus photographs and OCT scans^51–55^. Future work could explore application of deep learning systems to OCT-A images for evaluation of ocular and systemic complications of diabetes.

In conclusion, our study demonstrates that eyes with DR show global changes in retinal microvasculature, and that these changes can be quantified by semi-automated analysis of fundal photographs and OCT-A. In particular, CRAE and SVP CDI are significantly reduced in severe DR. OCT-A capillary parameters may be more closely associated with severe DR, but evaluations of the larger retinal vessels and the smaller capillaries are complementary, and both are important in global assessment of DR.

## Methods

### Study design

This was a prospectively-designed cross-sectional study involving 50 patients with type 2 diabetes mellitus recruited from the Diabetic Retinopathy Service at the Singapore National Eye Centre between October 2015 and June 2016. Patients were recruited with DR severity ranging from no DR to proliferative DR. Exclusion criteria included pregnancy, previous laser photocoagulation, concurrent retinal diseases other than DR, glaucoma, and media opacity adversely affecting fundus photography or OCT-A acquisition. This study was approved by the SingHealth Centralised Institutional Review Board, and was conducted in accordance with the tenets of the Declaration of Helsinki. All participants provided signed informed consent.

### Fundus photograph retinal arteriolar and venular parameters

All patients underwent logMAR VA measurement, and colour fundus photography including Early Treatment of Diabetic Retinopathy Study (ETDRS) standard fields 1 (optic disc-centered) and 2 (macula-centered). These were used to determine DR severity by trained graders who were masked to patient characteristics and retinal vascular parameters. DR severity grades included no DR, mild NPDR, moderate NPDR, severe NPDR, and proliferative DR, according to the International Clinical Diabetic Retinopathy Severity Scale^6^. No DR, mild NPDR and moderate NPDR were considered non-severe DR. Severe NPDR and proliferative DR were considered severe DR.

**Figure 1:**
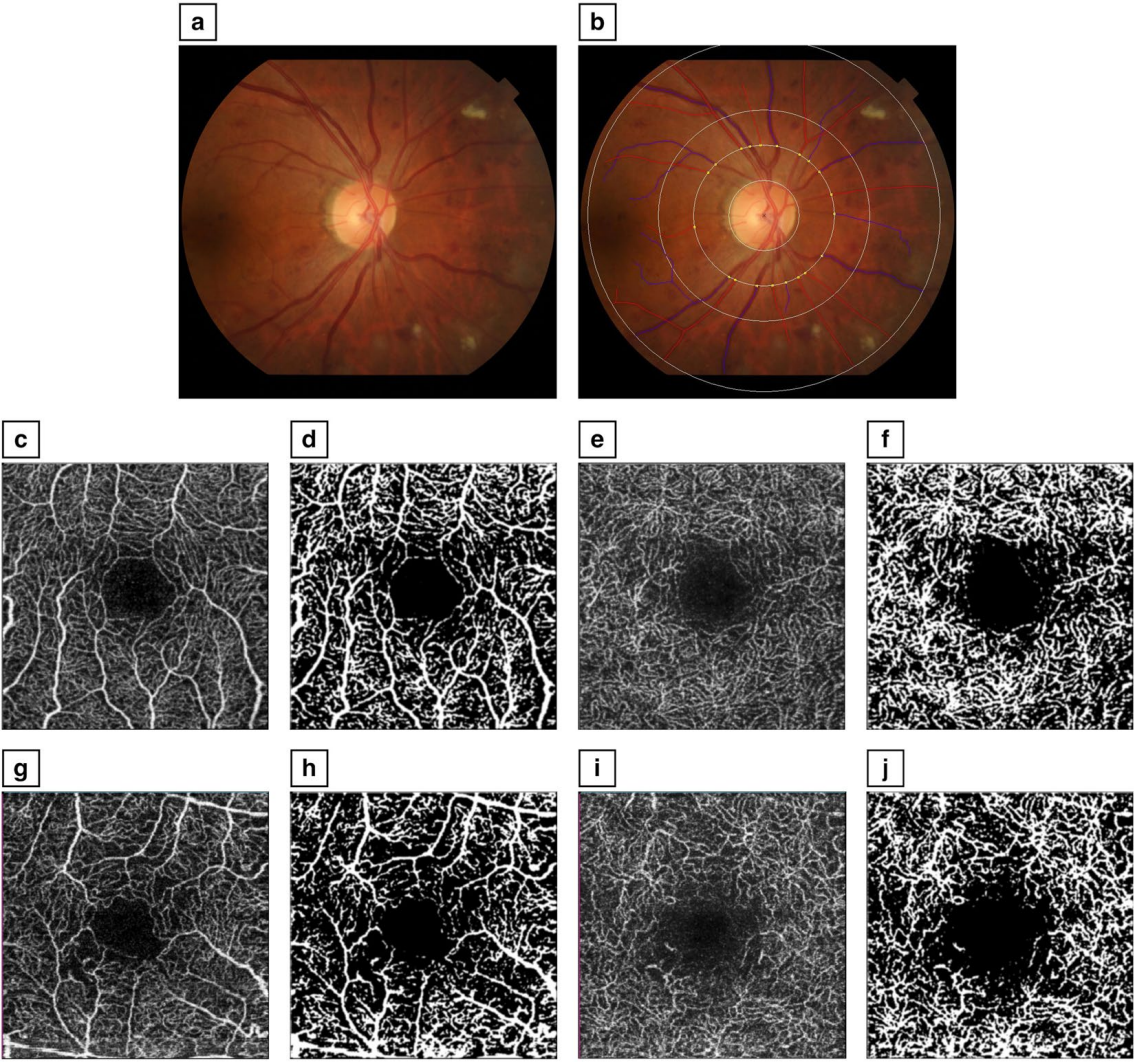
Measurement of retinal arteriolar, venular and capillary parameters. (**a**) Optic disc-centered fundus photograph of an eye with moderate non-proliferative diabetic retinopathy (DR). (**b**) The same photograph, using the Singapore I Vessel Assessment (SIVA) software, with the zone of measurement overlaid, and the skeletonized line tracing identifying arterioles (in red) and venules (in blue); This eye had a central retinal arteriolar equivalent (CRAE) of 143.6. (**c**) En face optical coherence tomography angiography (OCT-A) image of the superficial capillary vascular plexus (SVP) of an eye with no DR. (**d**) Binarized image of the SVP; This eye had an SVP capillary density index (CDI) of 0.38. (**e**) En face OCT-A image of the deep capillary vascular plexus (DVP) in the same eye. (**f**) Binarized image of the DVP; This eye had a DVP CDI of 0.38. (**g**) En face OCT-A image of the SVP of an eye with proliferative DR. (**h**) Binarized image of the SVP; This eye had an SVP CDI of 0.34. (**i**) En face OCT-A image of the DVP in the same eye. (**j**) Binarized image of the DVP; This eye had a DVP CDI of 0.35.

Optic disc-centered photographs were further analyzed by trained graders, using a semi-automated software program (Singapore I Vessel Assessment [SIVA], version 4.0). Graders were masked to patient characteristics and DR severity. Retinal arteriolar and venular parameters measured included CRAE, CRVE, arteriole-venule ratio (AVR), FD of all vessels (FDt), arterioles (FDa), venules (FDt), arteriolar and venular tortuosity, and branching angles and coefficients. All measurements were taken from the circumferential zone between 1- and 2-disc diameters from the optic disc margin (Fig. 1). CRAE and CRVE summarize the retinal arteriolar and venular caliber respectively, from the 6 largest vessels in the zone, and are derived based on the revised Knudtson-Parr-Hubbard formula^48,56,57^. AVR is the ratio of CRAE to CRVE^58^. FD is a measure of the complexity of the branching pattern of the retinal vascular tree^47,59^. This was calculated from the skeletonized line tracing using the box-counting method (Fig. 1)^48^. Branching angle was defined as the total angle between daughter branches, and branching coefficient was the width of the branches relative to the trunk^19^.

### OCT-A retinal capillary parameters

Swept-source OCT-A 3 × 3 mm macula scans were performed (DRI OCT Triton; Topcon Corporation, Tokyo, Japan). Images were processed with the angiography ratio analysis algorithm. Each en face OCT-A image was segmented into superficial (SVP) and deep (DVP) vascular plexuses (Fig. 1). For the SVP, the inner boundary was 3 μm beneath the internal limiting membrane, and the outer boundary was 15 μm beneath the IPL. For the DVP, the inner and outer boundaries were 15 μm and 70 μm beneath the IPL respectively. SVP and DVP images were analyzed using public domain software (ImageJ; National Institutes of Health, Bethesda, Maryland, USA) to obtain retinal capillary parameters, including CDI and capillary fractal dimension (FDc), as previously described^27^. In brief, images were converted to 8 bits and binarized, based on the Niblack thresholding technique (Fig. 1). A circle of radius 1.5 mm was centered on the fovea, and divided into 4 quadrants. The CDI of each quadrant was defined as the ratio of capillary luminal area to total area of the quadrant. CDI was averaged across all 4 quadrants. FDc was measured from the binarized images with Frangi vesselness, using the fractal box count method. Patients also had spectral domain optical coherence tomography (OCT) of the macula performed (Spectralis OCT; Heidelberg Engineering, Heidelberg, Germany), which was used to determine the presence of DME within the central 3 × 3 mm zone.

### Statistical analysis

Only the right eye of each patient was used for statistical analysis. Statistical analyses were performed using IBM SPSS Statistics version 24.0 (IBM Corporation, Armonk, New York, USA). Mean and SD were calculated for retinal vascular parameters at each DR severity level. These parameters were compared between DR severity levels, non-severe DR and severe DR groups, using the Wilcoxon-type test for trend and Mann-Whitney U test.

Associations between severe DR status, logMAR VA (independent variables) and retinal vascular parameters (dependent variables) were explored using linear regression models. DME manifests as oval dark areas in the DVP on OCT-A, which can be mistaken for areas of capillary non-perfusion, and affect OCT-A parameters^37^. Therefore, to account for this, DME was included as an additional independent variable in linear regression analyses. Univariate logistic regression analysis was also performed to associate vascular parameters with severe DR. McKelvey and Zavoina’s pseudo-R^2^ values were used to assess goodness-of-fit. Finally, as a secondary aim of the study, Spearman’s correlation analysis was performed to explore the association between OCT-A capillary parameters and SIVA arteriolar and venular parameters. P-values of < 0.05 were considered significant.

## Data Availability

The datasets generated and analyzed during the current study are available from the corresponding author on reasonable request.
